# Plasmonic black metals via radiation absorption by two-dimensional arrays of ultra-sharp convex grooves

**DOI:** 10.1038/srep06904

**Published:** 2014-11-04

**Authors:** Jonas Beermann, René L. Eriksen, Tobias Holmgaard, Kjeld Pedersen, Sergey I. Bozhevolnyi

**Affiliations:** 1 Department of Technology and Innovation, University of Southern Denmark, Niels Bohrs Allé 1, DK-5230 Odense M, Denmark; 2 Department of Physics and Nanotechnology, Aalborg University, Skjernvej 4A, DK-9220 Aalborg Øst, Denmark

## Abstract

Plasmonic black surfaces formed by two-dimensional arrays of ultra-sharp convex metal grooves, in which the incident radiation is converted into gap surface plasmon polaritons (GSPPs) and subsequently absorbed (via adiabatic nanofocusing), are fabricated and investigated experimentally for gold, nickel, and palladium, using scanning electron microscopy, optical microscopy, and reflection spectroscopy for their characterization. Absolute reflectivity spectra obtained for all fabricated arrays demonstrate very efficient and broadband absorption of unpolarized light exceeding the level of 95%, averaged over the investigated wavelength range of 400–985 nm. The highest averaged absorption level (~97%) is achieved with 250-nm-period arrays in palladium that also has the highest melting temperature (~1552°C), promising thereby potential applications for broadband absorption, e.g., within thermophotovoltaics. For one-dimensional arrays, GSPPs are excited only with the electric field polarized perpendicular to the groove orientation, resulting in 94–96% absorption of the appropriately polarized light for the arrays in nickel and palladium while featuring practically flat surface reflectivity spectra for the orthogonal polarization. The largest ratio (~10.7) between averaged reflectivities for orthogonal polarizations is achieved with the groove arrays in palladium, pointing thereby towards applications as broadband and low-dispersion linear polarizers operating in reflection, e.g., within ultra-fast optics.

Metal nanostructures and plasmonics in general offer numerous possibilities of new functional materials and advanced configurations, involving radiation guiding, manipulation, and nanofocusing beyond the diffraction limit^1^, bridging thereby freely propagating photonic modes with surface plasmon polaritons (SPPs) and nm-localized fields^2^ and improving also, e.g., photovoltaic devices^3^. Composite materials with selective absorption and thermal emission properties have also been suggested, including for example ordered arrays of gold-cross resonators placed on a dielectric spacer layer above a planar gold surface^4^, ordered arrays of metal elements combining two nano-scale split-rings^5^, arrays of ultra-thin silver resonant absorbers^6^, or random arrays of colloidal metal geometries or nanoantennas^7^. Tapered nanostructures, such as plasmonic V-grooves, involve strong SPP confinement to the groove bottom, for example, used when guiding SPPs along individual grooves^8^. One-dimensional (1D) gratings of plasmonic V-grooves were treated theoretically^9^ and local intensity enhancement at the bottom of individual V-grooves has been experimentally characterized and ascribed directly to gap SPP (GSPP) nanofocusing by the closed tapered shape^10^.

Recently, in order to realize efficient non-resonant (broadband) light absorption, we fabricated periodic (~250 nm) arrays of relatively deep (~500 nm) convex grooves in gold (with walls curving and meeting with vanishing small angle at the groove bottom), thereby harvesting and guiding on average ~96% of incident light (450–850 nm) toward the groove bottom by which it is gradually absorbed^11^. The underlying physical mechanism responsible for broadband light absorption in this configuration is related to radiation nanofocusing^12^,^13^,^14^, in which GSPPs (excited by scattering of the groove tops) propagate down toward the groove bottoms, being adiabatically squeezed and absorbed in ultra-sharp convex grooves (plasmonic black gold)^11^. In addition, due to the inherent polarization sensitivity of the GSPP excitation in grooves (polarization should be perpendicular to the groove)^15^, the 1D structures were very polarization sensitive, whereas two-dimensional (2D) arrays of crossing grooves showed high absorption for both in-plane polarizations, appearing dark also for unpolarized illumination (plasmonic black gold)^11^. Very recently, it has been demonstrated that the 1D arrays of ultra-sharp convex grooves in gold can function also as broadband polarizers (~600–800 nm) for reflection of ultra-short laser pulses due to practically zero (<1 fs) dispersive pulse stretching observed even with only 5–10 fs laser pulses^16^. Furthermore, 1D groove arrays were recently fabricated in nickel and investigated experimentally, exhibiting (for the polarization perpendicular to the groove orientation) only 5–8% reflectivity over the entire range of 400–1700 nm^17^ and offering thereby an excellent and relatively cheap (as compared to gold) way of producing broadband reflection polarizers for ultra-short laser pulses^16^.

In this work, we demonstrate experimentally plasmonic black nickel and palladium by ion-milling 2D arrays of ultra-sharp convex grooves, whose characteristics are investigated using scanning electron microscopy (SEM), optical microscopy, and reflection spectroscopy. Additionally, 1D groove arrays are fabricated and characterized, facilitating the comparison of gold, nickel and palladium for achieving efficient and broadband absorption of polarized and unpolarized light with 1D and 2D groove arrays, respectively. Application perspectives for these configurations along with the material selection are also discussed.

## Results

### 1D arrays

The black nickel and palladium characterized here were obtained with focused ion beam (FIB) written 400-nm-deep and 250-nm-periodic convex groove arrays. Due to gold's inherent less material absorption and therefore relatively deeper propagation toward the gold groove bottom, the black gold arrays needed slightly larger depth (450 nm) in order for these to also appear really black.

**Figure 1:**
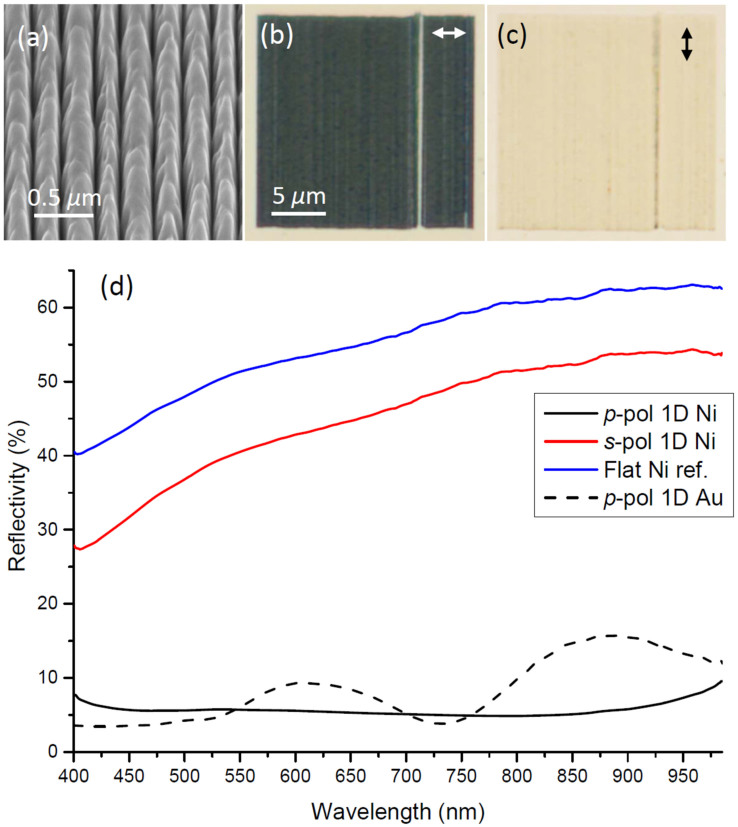
(a) SEM image of *1D black nickel* taken at 54 deg., along with (b)–(c) optical microscopy images obtained for the incident electric field polarization directions indicated by arrows, and (d) corresponding reflectivity spectra including a flat nickel reference and 1D black gold for comparison (Supplementary Fig. S1).

SEM images of 1D plasmonic black nickel reveal the overall 250-nm-period, although with some local variation between grooves, probably caused by nm-drift of the FIB between the multiple passes used to obtain the convex shape and ultra-sharp groove terminations at the bottom (Fig. 1a). The optical microscopy images of the 1D black nickel arrays exhibit the expected very large difference in reflection between the *p*-polarized (electric field perpendicular to the grooves) and *s*-polarized (electric field along the grooves) excitation direction indicated by arrows [Fig. 1(b) and (c)]. Overall, the array appears quite homogeneous apart from one clearly visible vertical line defect, most likely caused by consecutive FIB millings being displaced approximately half a period (~125 nm) for a single groove. Such a displacement leads to a broader and less deep groove, i.e. without the convex ultra-sharp shape and therefore exhibiting some reflection from the bottom for *p*-polarized excitation, whereas a more open non-ideal groove with some roughness at the bottom, leads to increased absorption and scattering for *s*-polarized excitation.

The obtained experimental reflection spectra from the 1D black nickel array reveal practically uniform reflectivity of ~6% over the wavelength range 400–985 nm for *p*-polarized light, whereas the reflectivity for *s*-polarized light is on average ~45%, increasing with wavelength and following that of the flat nickel reference, but consistently with ~10% lower reflectivity [Fig. 1(d)]. For comparison, the 1D plasmonic black gold *p*-polarized reflectivity exhibit some oscillations [Fig. 1(d), Supplementary Fig. S1], which has been related to combined interference effects of mutual phase differences in the remaining reflectivity from groove bottoms, caused by nm-variations in the groove depth, and non-ideal surface geometry of the array^17^.

**Figure 2:**
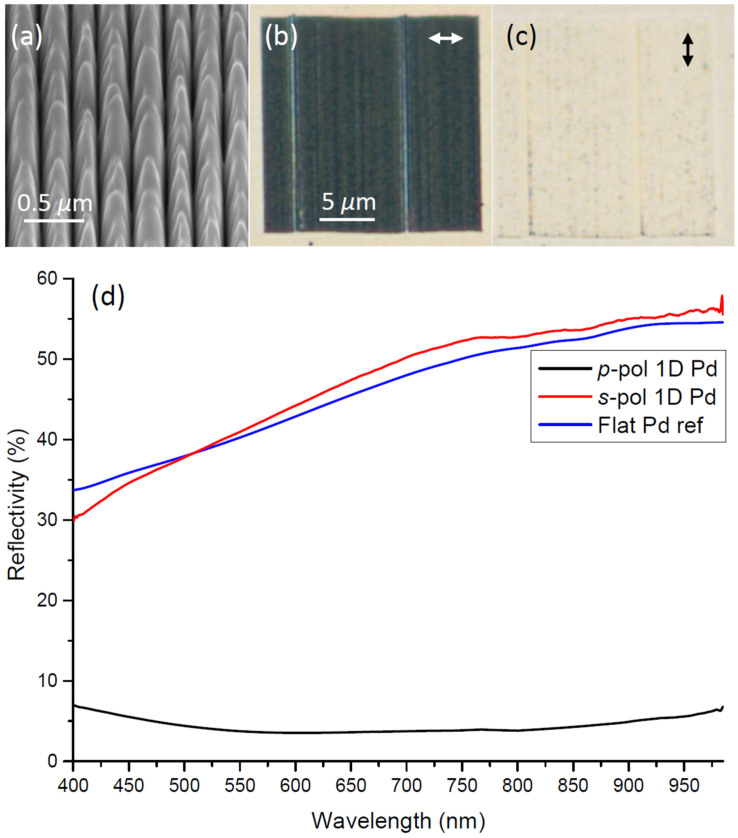
(a) SEM image of *1D black palladium* taken at 54 deg., along with (b)–(c) optical microscopy images obtained for the incident electric field polarization directions indicated by arrows, and (d) corresponding reflectivity spectra including a flat palladium reference.

SEM images of 1D *plasmonic black palladium*, appearing very similar to those of 1D black nickel, reveal the intended overall 250-nm-period and again some local variations probably caused by nm-drift of the FIB [Fig. 2(a)]. As expected, the optical microscopy images of 1D plasmonic black palladium arrays also exhibit very large differences between the *p*- and *s*-polarized excitation directions [Fig. 2(b) and (c)]. Again, a few line defects observed with *p*-polarized light [Fig. 2(b)] indicate sensitivity to nm-displacement of the FIB during consecutive millings. Interestingly, for *s*-polarized excitation the line defects in palladium are only weakly seen and not really distinguishable from the surrounding flat palladium background [Fig. 2(c)]. The obtained experimental reflection spectra from the 1D black palladium array reveal less than ~4% reflectivity over the wavelength range 550–850 nm for *p*-polarized light, whereas the reflectivity for *s*-polarized light is ~45% on average and increasing with wavelength [Fig. 2(d)].

For 1D black nickel the ~10% reduced reflectivity for *s*-polarized light compared to that of a flat nickel reference [Fig. 1(d)] is presumably due to the increased surface roughness introduced by the FIB structuring and is more or less expectable. However, it is interesting that for 1D black palladium we did actually not observe decreased reflectivity for *s*-polarized light compared to a flat palladium reference [Fig. 2(d)]. For palladium, the flat surface reflectivity obtained over the range 400–985 nm is ~5% less compared to flat nickel, but at the same time 1D black palladium arrays illuminated with *s*-polarized light exhibit reflectivity practically identical to the flat palladium reference [Fig. 2(d)]. Note that, theoretically the flat palladium reflectivity for normal incidence is in the range ~55–70%^18^, and even for angled incidence the flat palladium reference reflectivity should still be expected to be ~45–60% when qualitatively taking the influence of larger incidence angles into count. This indicates that our flat reference palladium surface possibly has increased roughness or has somehow been contaminated, and that the black palladium fabrication and subsequent SEM characterization maintained or likely even improved on the optical surface roughness and composition along the grooves. On the other hand, black nickel fabrication somehow produced slightly increased surface roughness and possible interruptions by material re-deposition into nm-scatterers along the groove tops, although this is difficult to elaborate directly from SEM images (Fig. 1a vs. Fig. 2a). The fact that the flat palladium reference reflectivity is nearly maintained with *s*-polarized illumination of the 1D black palladium array is clear also from the optical microscopy image, where array boundaries are barely seen [Fig. 2(c)].

### 2D arrays

**Figure 3:**
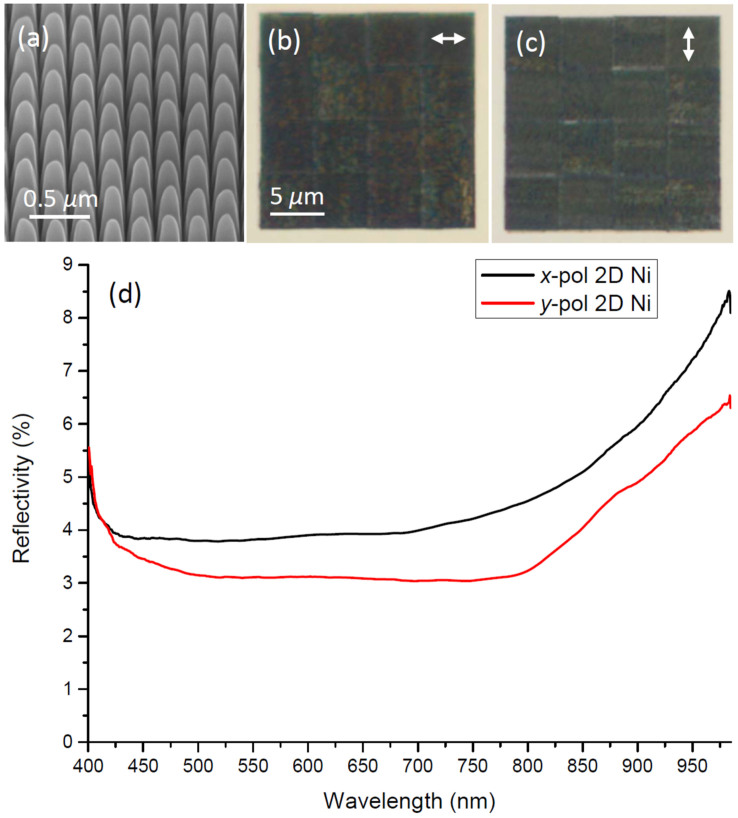
(a) SEM image of *2D black nickel* taken at 54 deg., along with (b)–(c) optical microscopy images obtained for the electric field polarization directions indicated by arrows, and (d) corresponding reflectivity spectra.

For two perpendicular line arrays of crossing convex grooves one obtains the so-called 2D plasmonic black metal with broadband absorption for both polarizations in the sample plane^11^. Note that previous issues with anisotropic absorption due to minor re-deposition along the groove direction milled first^11^, has now been avoided by improved FIB fabrication procedure and also due to the inherent higher absorption in nickel and palladium, which makes it relatively easier to obtain a high GSPP loss with these metals compared to gold (Supplementary Fig. S2), even with possibly less perfect groove terminations at the bottom. SEM images of the 2D black nickel array reveal very homogenous structures [Fig. 3(a)], which are also partly facilitated by stepwise fabrication of smaller more precise sub-squares. Polarized optical microscopy images of the 2D black nickel array display 16 stitched ~5 × 5 *μ*m^2^ sub-squares and the combined (20 × 20 *μ*m^2^) array is visually black for both polarizations [Fig. 3(b)–(c)]. Reflection spectroscopy of the 2D array also reveals the intended practically isotropic broadband absorption, i.e., with average reflectivity being ~4–5% in the wavelength range 400–985 nm [Fig. 3(d)].

**Figure 4:**
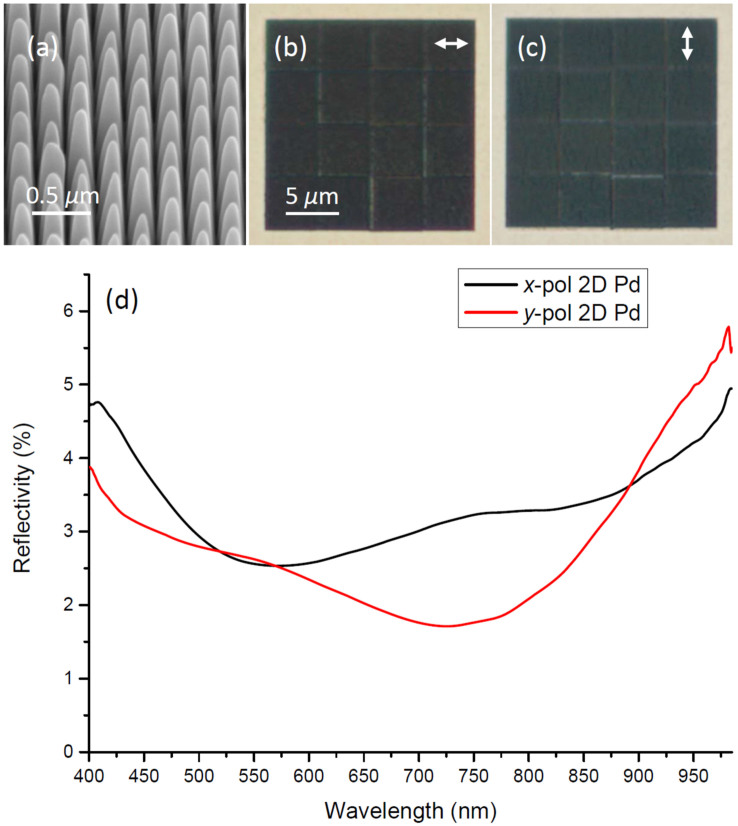
(a) SEM image of *2D black palladium* taken at 54 deg., along with (b)–(c) optical microscopy images obtained for the electric field polarization directions indicated by arrows, and (d) corresponding reflectivity spectra.

With 2D black palladium we also obtain quite homogenous structures [Fig. 4(a)], i.e., without the general roughness and imperfections observed along grooves in 1D structures [Fig. 2(a)]. Again this improved quality with 2D structures should partly be explained by the stepwise fabrication of smaller more precise sub-squares and the careful reduction of material re-deposition by consecutive FIB runs in perpendicular directions. However, comparing 2D black nickel, with 2D black palladium we obtained slightly sharper “domes” left in the areas between the crossing grooves [cf. Fig. 3(a) vs. Fig. 4(a)]. According to SEM images the domes with palladium are also to some extent less identical and a few are slightly misplaced or at an angle [Fig. 4(a)]. On the other hand, the sharper top-shape minimizes any flat surface left in the sample plane, which could lead to increased reflectivity^17^. The polarized optical microscopy images of the 2D black palladium reveal visually black arrays for both polarizations, practically without any scatterers showing up, except for faintly recognized less perfect junctions of the 16 stitched ~5 × 5 *μ*m^2^ sub-squares [Fig. 4(b)–(c)]. Note that for both nickel and palladium 2D arrays, the boundaries between stitched sub-squares are mainly visible with the electric field polarization being perpendicular to the boundary. Reflection spectroscopy of the 2D array also exhibits the intended broadband absorption, with average reflectivity level for both polarizations being only ~3% over the wavelength range 400–985 nm [Fig. 4(d)].

#### Comparison of 1- and 2D arrays

**Figure 5:**
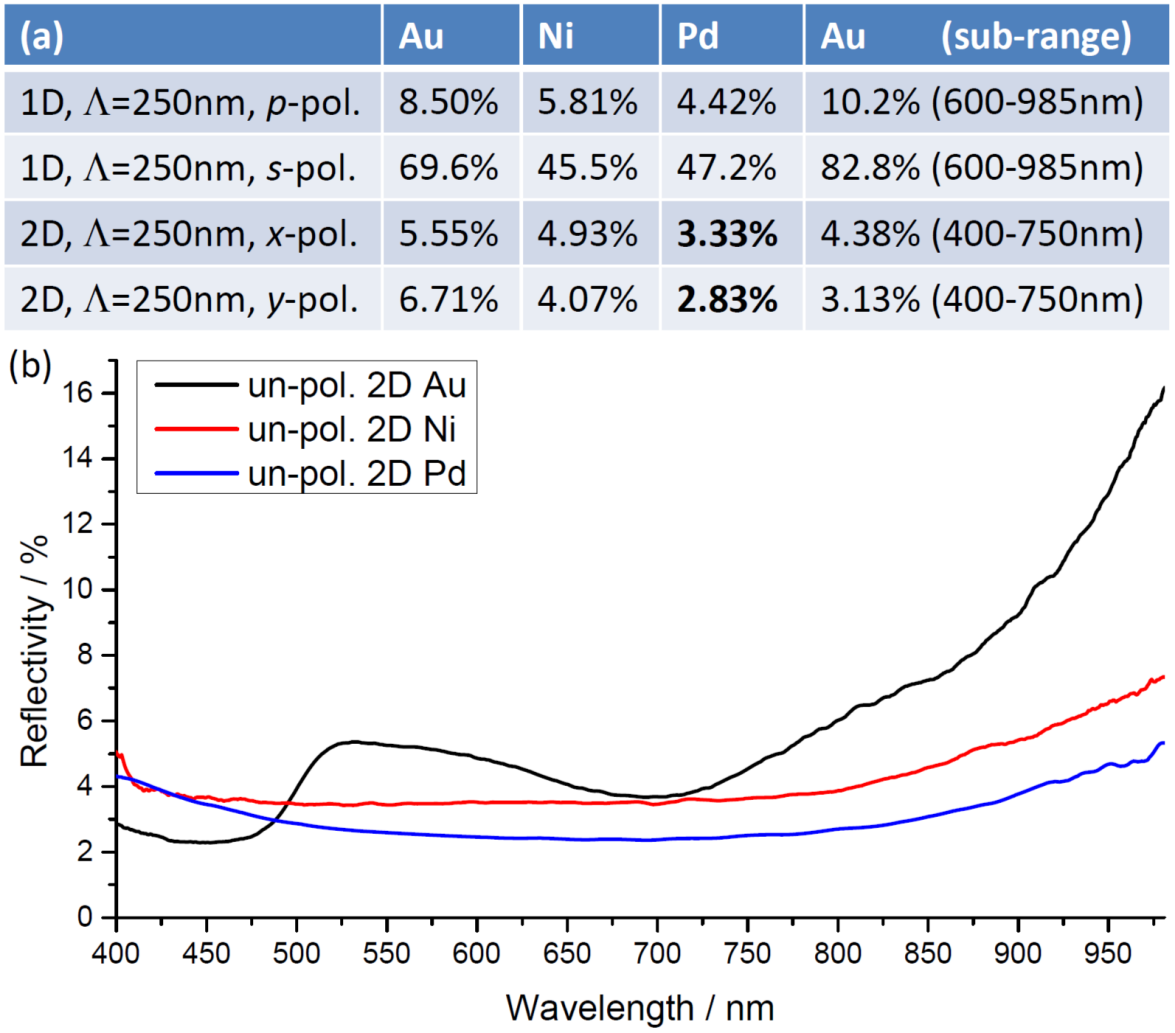
(a) Experimental polarized reflectivity levels for the 1- and 2D plasmonic black metals (Au, Ni, and Pd) averaged over the wavelength range 400–985 nm (773 CCD pixels) for each spectrum in Figs. 1,2,3,4, and Supplementary Figs. S1–S2. The last Au column is reflectivity averaged over the indicated sub-ranges.

We now summarize the obtained average reflectivity for each spectrum in Figs. 1,2,3,4, along with *unpolarized* reflectivity spectra of the 2D plasmonic black gold, nickel, and palladium [Fig. 5]. For the 1D structures the ratios between *p*- and *s*-polarized average reflectivity are around 1:8 for gold and nickel, and 1:10.7 for palladium [Fig. 5(a)]. This reflection anisotropy enables the recently demonstrated application of 1D structures as broadband linear polarizers operating in reflection and inducing negligible dispersive stretching of ultra-short (5–10 fs) laser pulses^16^. It is interesting to note that, even without the beneficial GSPP excitation and absorption, the inherent differences in *s*- and *p*- reflection coefficient dependences on angles of incidence (going through a minimum at pseudo Brewster angles for *p*-polarization, while increasing reflectivity for *s*-polarization, by Fresnel relations), along with the inclined surface geometry of the grooves, would already in itself (for some *effective* non-normal averaged angle of incidence) favor reflectivity of *s*-polarized versus *p*-polarized light. On the other hand, the refined 2D structures enable broadband and efficient absorption for both in-plane polarizations. The black nickel, and in particular black palladium, demonstrated experimentally here for the first time, exhibit practically homogenous light absorption of ~97% for unpolarized light measured in the wavelength range 400–985 nm [Fig. 5(b)], which can prove useful, e.g. within thermophotovoltaics. In this connection, any influence of the angle of incidence should also be considered. Note that the impressive low ~3% reflectivity for unpolarized light was obtained even with numerical aperture (NA) = 0.9, where the main part of any possible scattering from the dome-like tops would actually be re-collected by the objective. Comparing reflectivity from black gold obtained with various smaller NAs (down to 0.4) did not reveal any substantial differences^11^.

## Discussion

Experimentally we found that nickel and palladium are less sensitive than gold to the final sharpness of the groove bottom, which is in accordance with theoretical investigations since the propagation loss in these configurations is sufficient to practically prevent the excited GSPPs from reaching the groove bottoms^11^,^18^. For gold the propagation loss of GSPP decreases with longer wavelengths, and hence for the plasmonic black gold we observe higher reflection and some interference for the near-infrared wavelength range^17^, whereas the plasmonic black nickel and palladium exhibit practically flat spectra, also in the near-infrared range [Fig. 5]. For further improvements of broadband absorbers plasmonic black *chromium* could be considered as this has been predicted theoretically to give even lower reflections^18^. One should also mention improved possibilities with scanning helium ion-microscopes where the use of lighter ions for the imaging and FIB milling could possibly facilitate even better convex shape and ultra-sharp groove terminations at the bottom.

For applications as broadband absorbers methods for fabrication of large areas should be developed. Recent fabrication of related 2D plasmonic black metals (Ag, Au, and Al) in tunable *resonant* nanocavities using *template* fabrication techniques^19^ opens up a promising route for cheaper mass production of larger area black metal structured surfaces. Another concern with high-temperature applications is damage of the structures. The bottom of the adiabatic groove structure involves tiny nanogaps which might be quite sensitive to local damage. During evaluation of the local field intensity enhancements via two-photon luminescence (TPL) scanning optical microscopy^20^, we were able to permanently introduce some local damage to a 2D black gold structure using 3 mW 100 fs pulses focused to a 0.7 *μ*m spot size, leading to increased reflectivity and decreased TPL by the field enhancement at the groove bottom. For pulsed illumination we estimated a safe level in the range of 1 mW/*μ*m^2^, although this should not be directly extrapolated for larger areas, since the heat is not only dissipated into the underlying substrate, but also distributed very well in gold to regions outside the illumination spot. However, also for extended exposure of a 1D plasmonic black gold *polarizer* to 0.5 W, 10-fs-pulsed, 95 MHz repetition rate, 800 nm *p*-polarized radiation on a spot size ~50 *μ*m with ~75% absorption, no damage was noticed^16^. In addition, the higher melting point of nickel (~1453°C) and palladium (~1552°C), compared to gold (~1063°C), could facilitate increased operational temperature range of the black nickel / palladium devices.

Note that, the above mentioned damage observed with pulsed illumination might not have occurred with continuous illumination were the peak electric fields will be considerably reduced. If the nature of damage is purely thermal, then the structure would still suffer damage also for continuous illumination, whereas damage introduced via local breakdown by strong (peak) electric fields at the grove bottom would mainly occur for pulsed radiation.

Oxidation could be a concern when switching to configurations of less noble metals, like the much cheaper nickel or even proposed black chromium^18^ (melting point ~1857°C). Note that absolute reflectivity spectra of current black nickel samples did not show substantial differences after being stored in ambient air at room temperature even for several months. However, considerably elevated temperatures due to extensive light absorption and local heating could possibly cause increased long-term oxidation effects, which might influence the reflectivity spectra and remains to be investigated. On the other hand, for large areas the focus would be on improving both fabrication procedure *and* material costs. Currently, the palladium price is around 2/3 of the gold price, but actually palladium appears ~30 times rarer, so in this sense nickel or possibly chromium might be better alternatives. All in all, the necessary material properties and structures should be considered for each separate application, as a compromise between state of the art achievable broadband absorption (fabricated with FIB accuracy and at high costs), its stability (possible issues with oxidation as well as melting points), and larger areas with cheaper metals and possibly mass-production.

In conclusion, plasmonic black surfaces by arrays of ultra-sharp convex grooves in metals via GSPP excitation and subsequent adiabatic nanofocusing were compared experimentally for gold, nickel or palladium. We fabricated 1- and 2D groove arrays and employed SEM, optical microscopy, and reflection spectroscopy. We obtained absolute reflectivity spectra for each array and quantitatively compared levels averaged over the investigated wavelength range 400–985 nm. The 2D black nickel and black palladium exhibit average absorption of 95–97% for unpolarized light (400–985 nm). For 1D structures, GSPPs are only excited with electric fields perpendicular to grooves leading to 94–96% absorption, while practically maintaining flat surface reflectivity for electric fields along grooves. Plasmonic black palladium appears the so far most suitable configuration for achieving efficient and broadband light absorption by the use of groove geometries supporting adiabatic nanofocusing, promising diverse applications, e.g., within thermophotovoltaics (2D arrays) and ultra-fast optics (1D arrays).

## Methods

### Fabrication

Nickel and palladium films were deposited on a plasma cleaned Si substrate by e-beam deposition with a deposition rate of 1 Å/s until reaching a thickness of 800 nm. Gold films were deposited in a similar fashion and with the same deposition rate, but in this case by utilizing DC sputtering. The convex grooves were milled in the metal films by utilizing a FIB system, in which Ga+ ions with a current of 20 pA, 50 pA, or 100 pA, depending on material and desired depth, were scanned across the film thereby milling convex grooves. The ion beam was focused on the surface of the metal film and controlled by a lithography system. We obtained the convex groove shape tending toward infinitely narrow terminations at the bottom by decreasing the 1D groove periods until there were no flat sections between the grooves and simultaneously FIB milling as deep grooves as possible^11^,^17^. The 2D arrays were fabricated with the aim of achieving relatively low reflection anisotropy by FIB milling orthogonally oriented grooves in several passes, while changing the milling direction to be along *x, y, x, y*, and finally one pass along the *x*-axis with half the dose. We adapted this procedure in order to obtain ultra-sharp groove bottoms and reduce material re-deposition between grooves. The FIB milling was terminated once the top level of 2D arrays started to decrease below the flat metal surface, limiting thereby the groove depths. Furthermore, with the 2D arrays we divided the FIB writing into smaller adjacent 5 × 5 *μ*m^2^ sections, which then added up to form the final 20 × 20 *μ*m^2^ arrays. We use this approach to fabricate both 1D and 2D plasmonic black nickel and palladium arrays with 250 nm period and groove depths d ~ 400 nm. In addition, we compare with previously described 250-nm-periodic 1D black gold arrays of depths 450 nm and 2D black gold arrays of depths only 250 nm^11^ (Supplementary Figs. S1–S2.).

### Characterization

All SEM images of the black metal arrays were obtained at a tilt angle of 54 deg., corresponding to normal incidence of the ion beam.

The optical microscopy images were obtained in reflection, with ×100 objective (NA = 0.9) and polarized (along *x* or *y* direction), using halogen light illumination. The microscope images (2,080 × 1,544 pixels) were captured with a digital color camera (XC30, Olympus) and white balance calibrated.

Reflection properties of the fabricated arrays were studied using spatially resolved linear reflection spectroscopy^21^. The current reflection spectroscopy set-up included a microscope (BX51, Olympus) equipped with a halogen light source enabling spectroscopic investigations in the wavelength range 400−985 nm. The reflected light was collected in the backscattering configuration using an MPlanFL (Olympus) ×100 magnification, NA = 0.9, objective. The image area analyzed by the spectrometer was limited by an iris with a diameter of 1 mm resulting in a circular probing area with a diameter of 10 *μ*m. The state of polarization was controlled using linear film polarizers placed in the beam path just before the microscope objective. A fiber coupled grating spectrometer QE65000, Ocean optics was used in order to measure the linear reflectance in the wavelength range 400−985 nm. The measured reflectivity spectra were all normalized with a “perfectly” reflecting silver mirror. Reflectivity levels averaged over 400–985 nm (Fig. 5) were obtained from corresponding 773 pixels of the spectrometer CCD.

## Author Contributions

S.I.B. conceived the experiments and coordinated the work. T.H. and K.P. fabricated the groove arrays. T.H. performed the SEM characterization of the samples. J.B. and R.L.E. carried out the optical characterization. All authors participated in discussion of the results. J.B. prepared the figures and wrote the manuscript based on input from all authors.

## Supplementary Material

Supplementary Information Plasmonic black metals via radiation absorption by two-dimensional arrays of ultra-sharp convex grooves

## References

[b1] Gramotnev D. K. & Bozhevolnyi S. I. Plasmonics beyond the diffraction limit. Nat. Phot. 4, 83–91 (2010).

[b2] Schuller J. A. *et al.* Plasmonics for extreme light concentration and manipulation. Nat. Mater. 9, 193–204 (2010). 20168343 10.1038/nmat2630

[b3] Atwater H. A. & Polman A. Plasmonics for improved photovoltaic devices. Nat. Mater. 9, 205–213 (2010). 20168344 10.1038/nmat2629

[b4] Liu X. *et al.* Taming the Blackbody with Infrared Metamaterials as Selective Thermal Emitters. Phys. Rev. Lett. 107, 045901 (2011). 21867022 10.1103/PhysRevLett.107.045901

[b5] Landy N. I., Sajuyigbe S., Mock J. J., Smith D. R. & Padilla W. J. Perfect Metamaterial absorber. Phys. Rev. Lett. 100, 207402 (2008). 18518577 10.1103/PhysRevLett.100.207402

[b6] Aydin K., Ferry V. E., Briggs R. M. & Atwater H. A. Broadband polarization-independent resonant light absorption using ultra-thin plasmonic super absorbers. Nat. Commun. 2, 517 (2011). 22044996 10.1038/ncomms1528

[b7] Moreau A. *et al.* Controlled-reflectance surfaces with film-coupled colloidal nanoantennas. Nature 492, 86–89 (2012). 23222613 10.1038/nature11615 PMC3584706

[b8] Bozhevolnyi S. I., Volkov V. S., Devaux E., Laluet J.-Y. & Ebbesen T. W. Channel plasmon subwavelength waveguide components including interferometers and ring resonators. *Nature*. 440, 508–511 (2006). 10.1038/nature04594 16554814

[b9] Søndergaard T. & Bozhevolnyi S. I. Surface-plasmon polariton resonances in triangular-groove metal Gratings. *Phys*. Rev. B 80, 195407 (2009).

[b10] Søndergaard T. *et al.* Resonant plasmon nanofocusing by closed tapered gaps. *Nano Lett*. 10, 291–295 (2010). 10.1021/nl903563e 20028028

[b11] Søndergaard T. *et al.* Plasmonic black gold: efficient broadband nonresonant light absorption via nanofocusing in ultra-sharp convex grooves. Nature Commun. 3, 969 (2012). 22828629 10.1038/ncomms1976

[b12] Nerkararyan K. V. Superfocusing of a surface polariton in a wedge-like structure. Phys. Lett. A 237, 103–105 (1997).

[b13] Stockman M. I. Nanofocusing of optical energy in tapered plasmonic waveguides. Phys. Rev. Lett. 93, 137404 (2004). 15524758 10.1103/PhysRevLett.93.137404

[b14] Gramotnev D. K. Adiabatic nanofocusing of plasmons by sharp metallic grooves: geometrical optics Approach. J. Appl. Phys. 98, 104302 (2005).

[b15] Beermann J. *et al.* Polarization-resolved two-photon luminescence microscopy of V-groove arrays. Opt. Express 20, 654–662 (2012). 22274389 10.1364/OE.20.000654

[b16] Skovsen E. *et al.* Plasmonic black gold based broadband polarizers for ultra-short laser pulses. Appl. Phys. Lett. 103, 211102 (2013).

[b17] Beermann J. *et al.* Plasmonic black metals by broadband light absorption in ultra-sharp convex grooves. New J. Phys. 15, 073007 (2013).

[b18] Søndergaard T. & Bozhevolnyi S. I. Theoretical analysis of plasmonic black gold: periodic arrays of ultra-sharp grooves. New J. Phys. 15, 013034 (2013).

[b19] Bora M. *et al.* Plasmonic black metals in resonant nanocavities. Appl. Phys. Lett. 102, 251105 (2013).

[b20] Beermann J. & Bozhevolnyi S. I. Two-photon luminescence microscopy of field enhancement at gold nanoparticles. Phys. Stat. Sol. (c) 2, 3983–3987 (2005).

[b21] Hohenau A. *et al.* Spectroscopy and nonlinear microscopy of gold nanoparticle arrays on gold films. Phys. Rev. B 75, 085104 (2007).

