# Supplementary material for: Plasmonic black metals via radiation absorption by two-dimensional arrays of ultra-sharp convex grooves
Source: Sci Rep. 2014 Nov 4;4:6904. doi: 10.1038/srep06904 (PMC4219155; doi:10.1038/srep06904)
Supplement: Supplementary Information — Plasmonic black metals via radiation absorption by two-dimensional arrays of ultra-sharp convex grooves [file srep06904-s1.pdf]

SUPPLEMENTARY INFORMATION

Plasmonic black metals via radiation absorption by two-dimensional arrays of ultra-sharp convex grooves

Jonas Beermann[1*], René L. Eriksen[1], Tobias Holmgaard[2], Kjeld Pedersen[2] & Sergey I. Bozhevolnyi[1]

[1]Department of Technology and Innovation, University of Southern Denmark, Niels Bohrs Allé 1, DK-5230 Odense M, Denmark.

[2]Department of Physics and Nanotechnology, Aalborg University, Skjernvej 4A, DK-9220 Aalborg Øst, Denmark.

[*]corresponding author: job@iti.sdu.dk

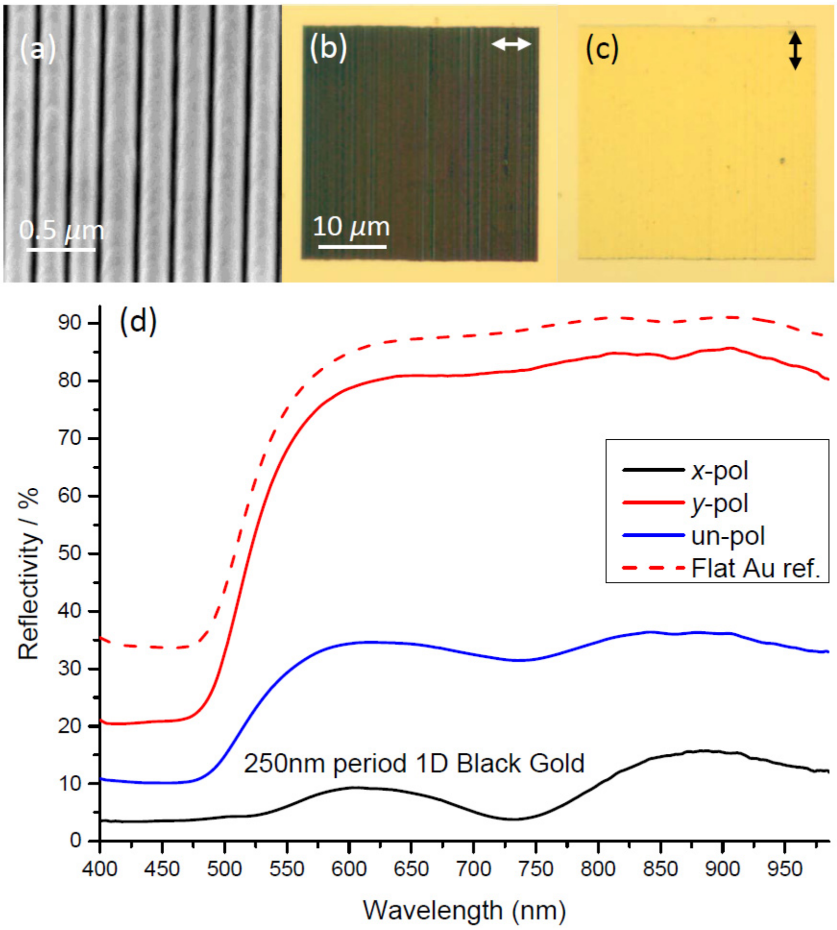

Figure S1. (a) SEM image of *1D black gold* along with (b)-(c) Optical microscopy images obtained for the incident electric field polarization directions indicated by arrows and (d) corresponding reflectivity spectra including a flat gold reference.

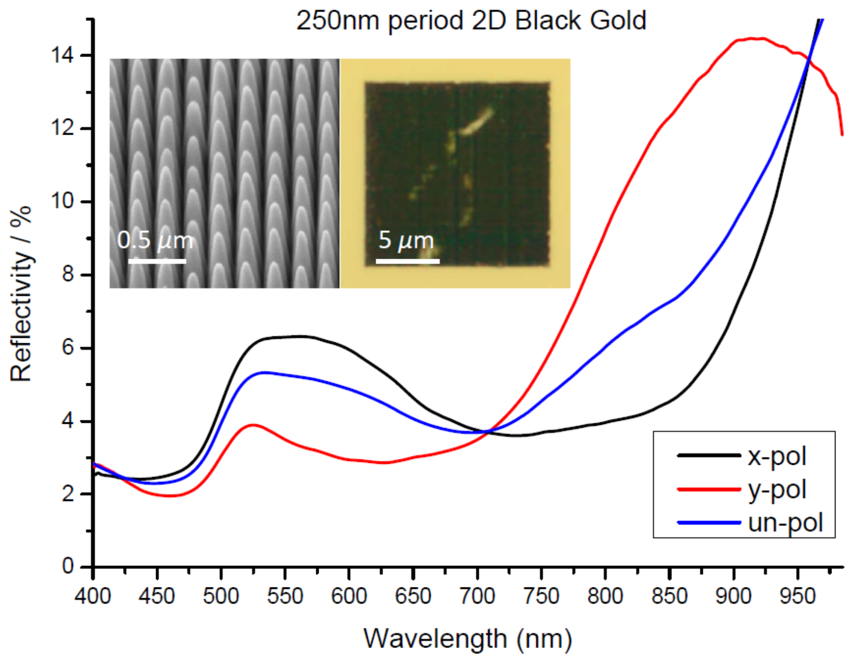

Figure S2. Reflectivity spectra of *2D black gold*, exhibiting some anisotropy due to material re-deposition by the previous FIB fabrication procedure[11]. Insert: SEM image taken at 54 deg. relative to normal incidence of the ion beam, along with un-polarized optical microscopy image of 2D black gold and smooth gold background, revealing minor post-fabrication damage to the structure.
